# Symbiosis Among *Naematelia aurantialba*, *Stereum hirsutum*, and Their Associated Microbiome in the Composition of a Cultivated Mushroom Complex JinEr

**DOI:** 10.3390/jof12010041

**Published:** 2026-01-04

**Authors:** Kaixuan Zhang, Yingli Cai, Xiaofei Shi, Zhuyue Yan, Qiuchen Huang, Jesus Perez-Moreno, Dong Liu, Zhenyan Yang, Chengmo Yang, Fuqiang Yu, Wei Liu

**Affiliations:** 1 Yunnan Key Laboratory for Fungal Diversity and Green Development & Yunnan International Joint Laboratory of Fungal Sustainable Utilization in South and Southeast Asia, The Germplasm Bank of Wild Species, Kunming Institute of Botany, Chinese Academy of Sciences, Kunming 650201, China; zhangkaixuan@mail.kib.ac.cn (K.Z.); yanzhuyue@mail.kib.ac.cn (Z.Y.); huangqiuchen0816@yeah.net (Q.H.); liudongc@mail.kib.ac.cn (D.L.); yangzhenyan@mail.kib.ac.cn (Z.Y.); yanglian2@mail.kib.ac.cn (C.Y.); 2 Key Laboratory of Chemistry in Ethnic Medicinal Resources, School of Ethnic Medicine, Yunnan Minzu University, Kunming 650500, China; loveylcai@163.com (Y.C.); shixiaofei@mail.kib.ac.cn (X.S.); 3 School of Biodiversity Conservation, Southwest Forestry University, Kunming 650224, China; 4 Colegio Postgraduados, Campus Montecillo, Edafología, Texcoco 56264, Mexico; jepemo@yahoo.com.mx

**Keywords:** microbiome, microbial community, JinEr mushroom, *Naematelia aurantialba*, *Stereum hirsutum*

## Abstract

The JinEr mushroom (“Golden Ear”), a globally rare edible and medicinal macrofungus, comprises a symbiotic complex formed by the symbiotic association of *Naematelia aurantialba* (*Tremellomycetes*) and *Stereum hirsutum* (*Agaricomycetes*). However, the interactions between these fungi and their associated microbiome remain poorly understood. This study employed high-throughput amplicon sequencing, in situ microbial isolation and culture, and microbial confrontation assays to analyze microbial diversity, community structure, and potential functional roles of the endomycotic bacterial community within JinEr basidiomata and its cultivation substrate. Molecular analysis confirmed the heterogenous composition of the basidiomata, revealing *N. aurantialba* constitutes less than 20% of the fungal biomass, while *S. hirsutum* predominates, accounting for approximately 80%. Endomycotic fungi accounted for 0.33% (relative abundance) of the fungal community. Prokaryotic analysis identified *Delftia* and *Sphingomonas* as the dominant endomycotic bacterial genera within basidiomata, comprising 85.42% of prokaryotic sequences. Endomycotic bacterial diversity differed significantly (*p* < 0.05) between basidiomata and substrate, indicating host-specific selection. Cultivation-based approaches yielded 140 culturable bacterial isolates (spanning four families and seven genera) from basidiomata core tissues. In vitro co-culture experiments demonstrated that eight representative bacterial strains exhibited compatible growth with both hosts, while one *Enterobacteriaceae* strain displayed antagonism towards them. These findings confirm that the heterogeneous JinEr basidiomata harbor a specific prokaryotic assemblage potentially engaged in putative symbiotic or commensal associations with the host fungi. This research advances the understanding of microbial ecology in this unique fungal complex and establishes a culture repository of associated bacteria. This collection facilitates subsequent screening for beneficial bacterial strains to enhance the JinEr cultivation system through the provision of symbiotic microorganisms.

## 1. Introduction

The basidiomata of macromycetes result from remarkable symbiotic interactions, which we have only begun to comprehend in greater depth through molecular and genomic tools available in recent decades. In the case of the basidioma formation of the mushroom, which is utilized in China mainly for its medicinal but also for its nutritive properties, designated jīn’ěr (金耳, literally “golden ear”), its formation is the consequence of an obligatory symbiosis comprising (i) *Naematelia aurantialba* (Schwein.) Burt (*Naemateliaceae*, *Tremellales*); (ii) *Stereum hirsutum* (Willd.) Pers. (*Stereaceae*, *Russulales*); and (iii) a currently uncharacterized microbiome of significant complexity. The “golden ear” or jīn’ěr (which will be referred to as JinEr hereafter) basidioma exhibits distinct morphological characteristics: a cerebriform, gelatinous to rubbery, bright, yellow-orange outer structure, contrasting with a fibrous, indurate, and grayish-white inner structure. This medicinally and culinarily significant mushroom has a broad distribution across Asia, Europe, North America and Oceania. Industrial-scale cultivation has been successfully established in China [1]. Polysaccharides derived from JinEr mushrooms have demonstrated substantial bioactive potential, exhibiting broad-spectrum therapeutic efficacy against diverse chronic pathologies, including neoplastic, metabolic (e.g., diabetes mellitus), cardiovascular, and inflammatory disorders [2]. JinEr mushroom has a long history in traditional Chinese medicine, and consequently its industrial cultivation has expanded dramatically during the last 20 years [3].

Owing to its inherent biological characteristics, the cultivation of JinEr mushroom necessitates the co-cultivation of both involved fungal species at a defined ratio within the spawn substrate [3]. Under both natural and artificial conditions, *N. aurantialba* is incapable of independent basidioma formation, whereas *S. hirsutum* completes its life cycle autonomously. Basidiomata of *S. hirsutum* are occasionally observed in proximity to naturally occurring JinEr basidiomata [4].

Research indicates that *N. aurantialba* engages in mycoparasitism of *S. hirsutum*. Mycoparasitism is operationally defined as a relationship wherein a fungus derives carbohydrates and other essential nutrients from another living fungus, during at least one stage of its life cycle [5]. Comparative genomic analyses revealed a reduced complement of Carbohydrate-Active enzyme (CAZyme) genes in the *N. aurantialba* genome relative to *S. hirsutum* and other saprophytic fungi [6]. This genomic deficiency suggests impaired capacity for autonomous nutrient acquisition, which is insufficient for completion of its life history. Furthermore, *N. aurantialba* develops haustorium-like structures within the hymenium and aerial mycelia when cultured on induction media [4]. Transmission electron microscopy (TEM) has demonstrated the presence of a minute membrane-bound channel at the interface between the haustorial apex of *N. aurantialba* and the hyphae of *S. hirsutum* [7], providing ultrastructural evidence for nanoscale fusion pore formation facilitating inter-fungal interaction [8]. However, the heterogeneous nature of the JinEr basidioma suggests functional contributions of *S. hirsutum* that extend beyond mere nutritional provisioning. The precise mechanisms governing this complex symbiotic interaction warrant further investigation.

During the artificial cultivation of JinEr mushroom, spawn development typically occurs on sterilized substrates. Nevertheless, some bacterial strains are consistently isolated, under axenic conditions, from tissues of cultivated JinEr basidiomata. This observation suggests the potential involvement of specific microbial communities in the growth and development of JinEr basidiomata. Analogous to plant and animal microbiomes, which are known to contribute to the ‘extended phenotype’ of their hosts, fungal microbiomes are recognized to influence host biology [9]. Microorganisms are ubiquitously associated with macrofungi, colonizing the mycelium, basidiomata, and even spores of them [10,11,12]. Current research indicates that mushroom-inhabiting bacteria (MIB) exhibit diverse functional roles, including, but not limited to, facilitation of basidioma morphogenesis, enhancement of sporulation efficiency, and dispersal [13]; biosynthesis of volatile organic compounds contributing to aroma [14]; secretion of growth-regulating metabolites [15]; and suppression of phytopathogens [16]. Host factors may exert a stronger influence on associated microbial community structure than environmental conditions. Under selective pressures or suboptimal environments, bacteria exhibit specificity for colonization of compatible macrofungal hosts [17]. Conversely, macrofungi selectively recruit bacterial taxa into their mycelium and basidiomata. For instance, *Rhizobium* spp. are frequently endosymbionts within species belonging to the genus *Cantharellus* [18] and *Laccaria* [19]. In cultivatable species (e.g., *Agaricus bisporus*), significant temporal shifts in substrate microbial community composition and relative abundance occur throughout production cycles [20,21]. Characterizing the structure and diversity of these microbial communities is therefore fundamental for resource discovery, functional elucidation, and understanding host-microbial interactions. However, the microbiome associated with JinEr basidiomata remains so far underexplored.

This study aims to investigate the endomycotic microbial communities within the JinEr mushroom cultivation system using an integrated approach. The specific objectives are (1) to characterize the composition, diversity, and distribution of fungal and bacterial communities across 17 anatomical compartments of the cultivation substrates and basidiomata via high-throughput amplicon sequencing, and to predict their potential ecological functions through comparative bioinformatic analyses; and (2) to isolate and identify culturable endomycotic bacteria, select representative strains based on phylogenetic analysis for co-culture experiments with the host fungus, and preliminarily assess their interactions to establish a foundation for screening beneficial microbial agents.

## 2. Materials and Methods

### 2.1. Sample Collection and Processing Methodology

Mature basidiomata of JinEr, along with their associated cultivation substrates contained within bags were obtained from a modern JinEr cultivation facility (Jincheng, China), and pure cultivation of *N. aurantialba* and *S. hirsutum* were obtained from Anhui Qigang Agricultural Development Co., Ltd. (Jieshou, China). To minimize the risk of microbial contamination, three representative mature basidiomata, each along with its substrate kept in the original cultivation bag, were selected for subsequent compartmental isolation. Each basidioma and its corresponding substrate constituted a discrete sample unit.

Sample collection was performed using sterile latex gloves to prevent potential exogenous contamination. Selected samples were individually placed into sterile sampling bags and transported to the laboratory under refrigerated conditions at 4 °C within a 12-h timeframe using insulated containers with ice packs. Prior to internal tissue processing, the external surfaces of all samples were subjected to decontamination with 75% (*v*/*v*) ethanol and subsequently were dried using sterile absorbent paper.

**Figure 1:**
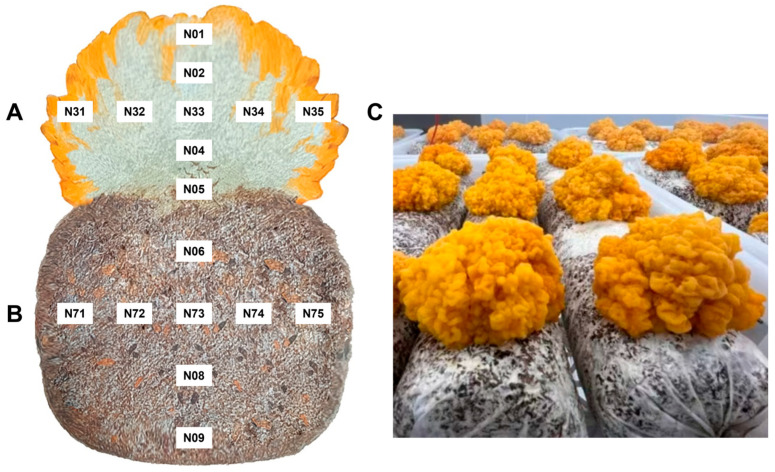
Schematic cross-sectional representation of the JinEr mushroom cultivation system. (**A**) Depicts the mature basidiome (N01, N02, N31, N32, N33, N34, N35, N04, N05); and (**B**) illustrates the substrate contained within the cultivation bag (N06, N71, N72, N73, N74, N75, N08, N09). (**C**) Photograph of JinEr mushrooms under industrial cultivation. Sampling locations are denoted by alphanumeric codes, with nine points within the basidiome and eight points distributed throughout the substrate.

Longitudinal bisection of each basidioma and its corresponding substrate was conducted under aseptic conditions employing a sterile scalpel. From each bisected section, a total of 17 internal tissue and substrate compartments were systematically sampled at defined vertical and horizontal positions, including nine across the basidiome and eight within the substrate matrix (Figure 1). The selection of 17 compartments were designed to capture microbial communities across a continuous spatial and anatomical gradient of the cultivation system, encompassing: (i) the substrate (foundational nutritional environment); (ii) the substrate-basidioma interface (critical interaction zone); and (iii) distinct internal basidioma tissues (focal point for endomycotic analysis). This design thus enables the assessment of microbial distribution in relation to physical location while simultaneously providing a holistic understanding of the heterogeneous distribution of *N. aurantialba* and *S. hirsutum* within the fruiting body and substrate.

Each excised tissue fragment, measuring approximately 1 cm^3^, was assigned a unique identification code. Sampling is performed at the same site on each sample, meaning that three independent biological replicate samples were collected from each sampling location. Each excised tissue fragment was immediately transferred into a sterile 5 mL polypropylene centrifuge tube. Samples were processed directly for endomycotic isolation or cryopreserved at −80 °C for subsequent microbial community analysis.

### 2.2. DNA Extraction and PCR Amplification

Fifty-one samples collected from 17 sampling points were subjected to microbial community analysis. Samples were homogenized and pulverized using an MM400 Mixer Mill (Retsch, Germany). Genomic DNA was subsequently extracted using the PowerSoil DNA Kit (MoBio Laboratories, Carlsbad, CA, USA; Cat. No. 12888). PCR amplification of the bacterial 16S rRNA genes V3–V4 region was performed using the forward primer 338F (5′-ACTCCTACGGGAGGCAGCA-3′) and the reverse primer 806R (5′-GGACTACHVGGGTWTCTAAT-3′) [22], Sample-specific 7 bp barcodes were incorporated into the primers for multiplex sequencing. The PCR components contained 5 μL of buffer (5 ×), 0.25 μL of Fast pfu DNA Polymerase (5 U/μL), 2 μL (2.5 mM) of dNTPs, 1 μL (10 μM) of each Forward and Reverse primer, 1 μL of DNA Template, and 14.75 μL of ddH_2_O. Thermal cycling consisted of initial denaturation at 98 °C for 5 min, followed by 25 cycles consisting of denaturation at 98 °C for 30 s, annealing at 53 °C for 30 s, and extension at 72 °C for 45 s, with a final extension of 5 min at 72 °C. For fungal community analysis, the internal transcribed spacer 1 (ITS1) region was amplified using a pair of primers: the forward primers ITS1F (5′-GGAAGTAAAAGTCGTAACAAGG-3′) and the reverse primer ITS2R (5′-GCTGCGTTCTTCATCGATGC-3′) [23]. Sample-specific 7 bp barcodes were incorporated into the primers for multiplex sequencing. The PCR mixture and volumes were identical to those used for bacteria, except for the annealing temperature (55 °C) and cycle number (30 cycles). The bacterial 16S and fungal ITS1 regions were amplified in separate, independent PCR reactions to optimize cycling conditions for each target.

Resulting PCR amplicons were purified using a Gel Extraction Kit (OMEGA BioTek, Doraville, GA, USA) and quantified with a NanoDrop 2000 (Thermo Fisher Scientific, Wilmington, DE, USA). Equimolar amounts of each purified amplicon were pooled based on quantification results to construct the sequencing library using the Illumina TruSeq Nano DNA LT Library Prep Kit (Illumina, San Diego, CA, USA). Library fragment size distribution was assessed using an Agilent 2100 Bioanalyzer system with the Agilent High Sensitivity DNA Kit (Agilent Technologies, Santa Clara, CA, USA). Qualified libraries should exhibit a single peak with no adapters. Library concentration was quantified using the Quant-iT^™^ PicoGreen^®^ dsDNA Assay Kit (Thermo Fisher Scientific, Waltham, MA, USA) on a QuantiFluor^®^ fluorescence measurement system (Promega Corporation, Madison, WI, USA). Libraries with a calculated concentration of ≥2 nM were considered qualified for subsequent sequencing. The pooled sequencing libraries were subjected to paired-end sequencing (2× 250 bp) on the Illumina NovaSeq 6000 platform (Personalbio Biotech Corporation, Shanghai, China). All raw sequencing data have been deposited in the NCBI BioProject under the accession number PRJNA1204465.

### 2.3. Microbial Community Diversity Analysis and Functional Prediction

Raw sequencing reads were processed and analyzed using the QIIME 2 pipeline. Processing steps included quality trimming, demultiplexing based on sample-specific barcodes, and removal of chimeric sequences and singletons. High-quality reads were clustered into amplicon sequence variants (ASVs) at a similarity threshold of ≥99%. Taxonomic assignment of bacterial ASVs were performed against the Greengenes database (for 16S rRNA) [20], while fungal ASVs were assigned using the UNITE database (for ITS) [24,25].

A one-way analysis of variance (ANOVA) with Tukey’s HSD test was conducted to compare bacterial and fungal abundance (both species and gene copy numbers), richness (Chao1 and observed species), and diversity (Shannon and Simpson indices) among samples of different compartments (basidiomata and substrates) [26,27]. Core and unique microbial taxa within compartments were identified based on ASVs and visualized using a petal diagram. Keystone taxa were identified through co-occurrence network analysis that was constructed from a bacterial correlation matrix. Highly interconnected taxa within the network were grouped into functional modules. Hub connectors within modules were determined based on topological roles, where module connectivity values exceeding 0.62 were considered indicative of significant modular structure, consistent with previous recommendations [28,29].

The functional potential of the bacterial communities was inferred from the 16S rRNA gene sequence data using predictive tools. Metabolic pathway predictions were generated using the MetaCyc database (available at https://metacyc.org/, accessed on 20 August 2024). Putative ecological functions were predicted using the Functional Annotation of Prokaryotic Taxa (FAPROTAX) database (available at http://www.loucalab.com/archive/FAPROTAX, accessed on 22 August 2024).

### 2.4. Isolation and Characterization of Culturable Endomycotic Bacteria

Basidioma tissue cores of measuring approximately 1 cm^3^ were aseptically excised and transferred to a sterile mortar. Tissue homogenization was performed via mechanical disruption with a sterile pestle after adding sterile distilled water. The resultant homogenate was filtered through a sterile 5 µm pore membrane to remove particulate debris and subjected to serial 10-fold dilution in sterile phosphate-buffered saline (PBS). Aliquots (100 μL) of appropriate dilutions were spread-plated onto the following solid media: Potato dextrose agar (PDA) [12 g/L potato infusion, 20 g/L dextrose, 14 g/L agar]; Reasoner’s 2A agar (R_2_A) [0.5 g/L trypton, 0.5 g/L yeast extract, 0.5 g/L casein and hydrolysate, 0.5 g/L glucose, 0.5 g/L starch from potato soluble, 0.3 g/L K_2_HPO_4_, 0.05 g/L MgSO_4_·7H_2_O, 0.5 g/L pyruvic acid sodium salt, Agar 14 g/L agar]; tryptic soy agar (TSA) [17 g/L trypton, 2.5 g/L glucose, 3 g/L soy Peptone, 5 g/L NaCl, 2.5 g/L K_2_HPO_4_, 14 g/L Agar]; and Luria–Bertani agar (LBA) [10 g/L tryptone, 5 g/L yeast extract, 10 g/L NaCl, 14 g/L agar]. Plates were incubated aerobically at 28 °C. Discrete colonies with distinct morphological characteristics were purified by successive quadrant streaking on fresh LBA to obtain axenic cultures. Pure isolates were transferred to LBA slants and stored at 4 °C for preservation.

For molecular identification, single colonies were inoculated into 1 mL of LB broth in 1.5 mL sterile centrifuge tubes and incubated with agitation (120 rpm) at 28 °C for 24 h. Transfer 20 μL of bacterial LB culture into a 200 μL sterile centrifuge tube. Add 100 μL PCR lysis buffer and 1 μL proteinase K (20 mg/mL) [Accurate Biotechnology (Hunan) Co., Ltd.; Code NO. AG12306], then mix thoroughly. Incubate at 60 °C for 5 min, followed by 98 °C for 2 min. Centrifuge at room temperature to pellet insoluble material to the tube bottom. Transfer the supernatant to a fresh centrifuge tube; store on ice or at 4 °C for later use. Bacterial genomic DNA was extracted from 2 µL of culture supernatant. Amplification of the 16S rRNA gene was performed in 25 µL reaction volumes containing: 12.5 µL 2× PCR Master Mix, 0.75 µL of a pair (27F: 5′-AGAGTTTGATCCTGGCTCAG-3′; 1492R: 5′-TACGGCTACCTTGTTACGACTT-3′), 2 µL of supernatant (template DNA), and 9.5 µL of ddH_2_O. The PCR amplification protocol included an initial denaturation step at 95 °C for 5 min, followed by 30 cycles of denaturation at 94 °C for 30 s, annealing at 55 °C for 30 s, and elongation at 72 °C for 1 min.

Amplicons were verified by electrophoresis, purified and subjected to bidirectional Sanger sequencing by Tsingke Biotech Corporation (Beijing, China). Raw sequences underwent quality trimming and assembly using CodonCode Aligner v10.0.02. Taxonomic affiliation was determined via BLASTn (2.16.0) analysis against the NCBI GenBank database. Phylogenetic reconstruction was performed using IQ-TREE v2.2.0 under the GTR + G + I model with 1000 ultrafast bootstrap replicates, following multiple sequence alignment with MUSCLE v3.8.31.

### 2.5. Antagonistic Assay Between Endomycotic Bacteria and Host

Based on phylogenetic analysis of the isolated endomycotic bacteria, representative strains from major, phylogenetically distinct clades were selected for in vitro antagonism assessment against their respective host fungi (*N. aurantialba* and *S. hirsutum*) using dual culture confrontation assays.

For *N. aurantialba*, activate it on PDA medium in a 6 cm diameter plate. An agar disk with mycelium (20 mm length × 2 mm diameter) was aseptically excised from an activated N. *aurantialba* mycelial colony used sterile cork borer and inoculated at the center of the plate. Selected endomycotic bacteria strains were subsequently inoculated in a cruciform pattern at equal distances (30 mm) from the central fungal inoculum. Monoculture controls for each bacterial strain and *N. aurantialba* were set up on separate PDA plates of the same type. All plates were incubated at 24 °C for 7 days. Antagonistic interactions were evaluated by examining growth patterns at the interaction zones. The absence of discernible growth inhibition or morphological alterations, for example, hyphal lysis or shrinkage, in either organism at the interaction insurface was considered no antagonism. An activated *S. hirsutum* mycelial plug (5 mm diameter × 2 mm thickness) was inoculated on one end of a 6 cm diameter PDA plate. The selected endomycotic bacterial strains were inoculated on the diametrically opposite end of the same plate. Corresponding monoculture controls for bacteria and *S. hirsutum* were prepared. Plates were incubated at 24 °C for 7 days. Antagonism was assessed by comparing fungal growth towards the bacterial inoculum with that in the control plate (growth away from the bacteria). Uninhibited radial growth of *S. hirsutum*, comparable to that in the control, was classified as no antagonism.

### 2.6. Statistical Analysis and Visualization

QIIME2 (v2019.4) was employed to characterize taxonomic composition and quantify alpha diversity metrics of symbiotic microbial communities, with visualization integrated into the workflow. Core bacterial taxa were identified and visualized via Venn diagrams using Origin 2024, while bacterial community co-occurrence networks were analyzed and visualized in R software (v4.3.1). PICRUSt2 (v2.5.1) was used to predict functional potential of symbiotic bacterial and fungal communities against the MetaCyc pathway database (v26.0), followed by functional profile visualization. Phylogenetic trees were constructed in MEGA11 using the maximum likelihood (ML) method (1000 bootstrap replicates for branch support) and refined for visualization on the ChiPlot platform (available at https://www.chiplot.online/, accessed on 15 September 2024).

## 3. Results

### 3.1. Composition of Bacterial and Fungal Taxa

Following quality filtering, removal of singletons, and chimeric sequences, a total of 51 samples yielded high-quality 16S rRNA gene amplicon sequences (ranging from 2 to 2,960,375 sequences, mean = 53,834). Bacterial taxonomic assignment revealed 32 phyla, 73 classes, 167 orders, 299 families, 638 genera. A total of 1109 ASVs were obtained, comprising 666 ASVs classified to known species and 443 ASVs classified as virtual taxa at the species level (Table S1). Similarly, the filtered fungal ITS sequences ranged from 2 to 5,063,382, mean = 32,827, and revealed 10 fungal phyla, 31 classes, 72 orders, 154 families, 253 genera, and a total of 359 fungal ASVs. Among these, 296 ASVs were identified as known species, while 63 ASVs represented virtual taxa at the species level (Table S2). Rarefaction analysis confirmed adequate sequencing depth for comprehensive microbial community characterization (Figure S1). ASV-based analysis revealed Proteobacteria as the dominant bacterial phylum, comprising a mean relative abundance of 92.98% across all samples. Substantially lower abundances were observed for Firmicutes (3.43%), Actinobacteriota (1.80%), and Bacteroidetes (1.47%). The remaining phyla collectively constituted 0.33% of total sequence abundance (Table S3).

**Figure 2:**
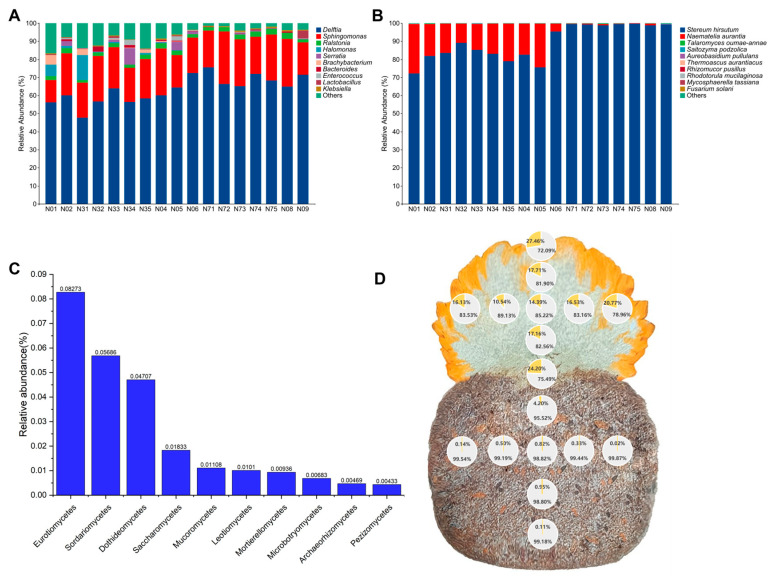
(**A**) Relative abundance of top 10 endomycotic bacterial genera. (**B**) Relative abundance of top 10 fungal species. (**C**) Relative abundance of top 10 endomycotic fungal classes excluding host fungi. (**D**) Relative abundance proportions of *Naematelia aurantialba* and *Stereum hirsutum* within a cross-section of the JinEr mushroom cultivation system. Unclassified or unidentified genera (**A**) or species (**B**) were aggregated as “Others”. Values represent means of three replicates.

**Figure 3:**
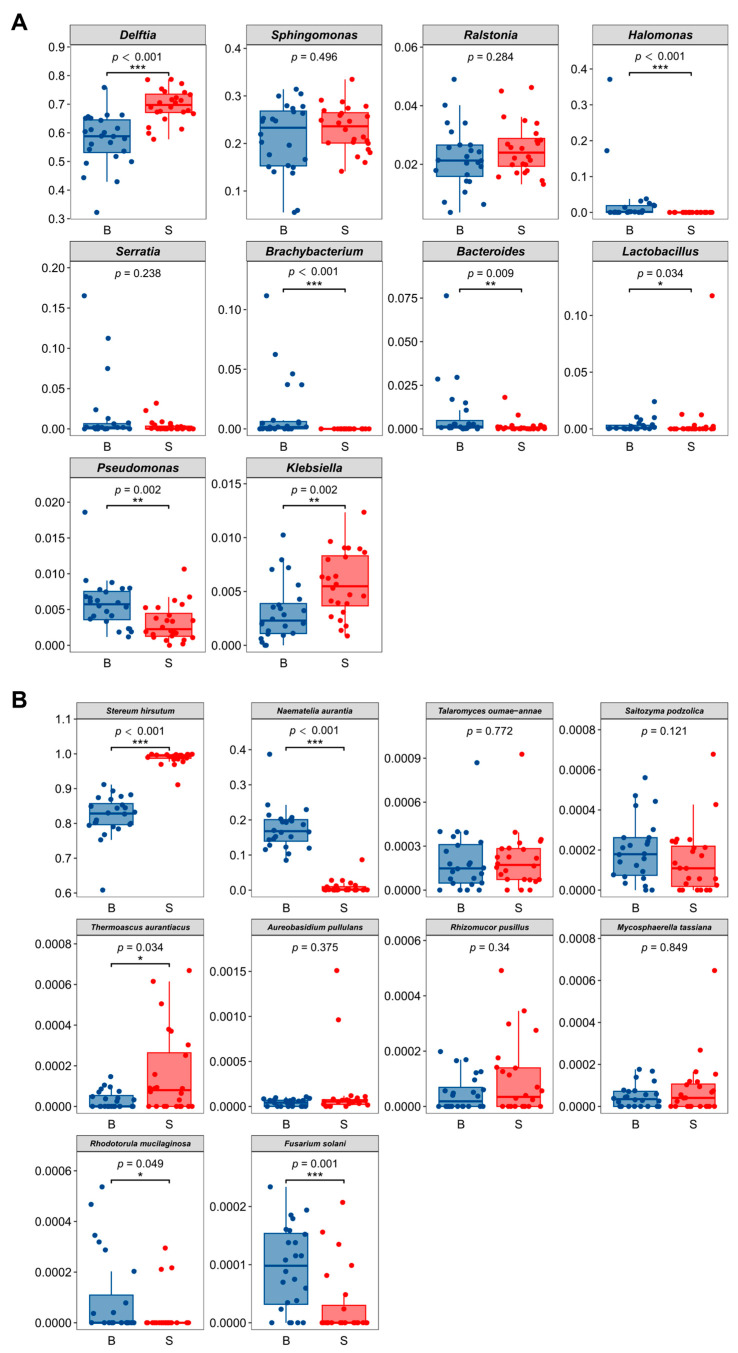
Comparative analysis of relative abundance differences for the top 10 endomycotic bacterial genera (**A**) and endomycotic fungal species (**B**) inhabiting the basidioma versus the substrate of JinEr mushroom cultivation system. Differences in relative abundance distributions were assessed using the Shannon diversity index. Horizontal lines above boxplots denote statistically significant pairwise comparisons by Student’s *t*-test. Asterisks indicate the significance level of the mean differences between groups: *** *p* < 0.001; ** *p* < 0.01; * *p* < 0.05. B: basidioma, includes 8 compartments (sampling points N01, N02, N31, N32, N33, N34, N35, N04); and S: substrate, includes 8 compartments (sampling points N06, N71, N72, N73, N74, N75, N08, N09) as illustrated in Figure 1 and Figure 2.

At the bacterial genus level, *Delftia* (63.51%) and *Sphingomonas* (21.91%) were the most abundant taxa. Other genera exhibited lower abundances: *Ralstonia* (2.37%), *Halomonas* (1.39%), *Serratia* (1.28%), *Brachybacterium* (0.64%), *Bacteroides* (0.50%), *Enterococcus* (0.48%), *Lactobacillus* (0.46%), and *Klebsiella* (0.44%) (Table S4). Significant inter-sample variation was observed in the abundance of these genera. For example, *Delftia* abundance ranged from 47.67% (sample N31) to 75.46% (sample N71), while *Halomonas* abundance ranged from 0% (sample N71) to 13.65% (sample N31) (Figure 2A). *Sphingomonas* abundance ranged from 12.36% (sample N01) to 29.07% (sample N72) (Figure 2A, Table S5). Furthermore, *Delftia* relative abundance was significantly higher (*p* < 0.05) in basidiomata compared with the substrate (Figure 3A). No significant difference in relative abundance was detected for *Sphingomonas.* Among the less abundant genera, six (*Halomonas*, *Brachybacterium*, *Bacteroides*, *Lactobacillus*, *Pseudomonas*, and *Klebsiella*) exhibited significant differences in relative abundance between the basidiomata and the substrate (Figure 3A).

Fungal communities were predominantly comprising Agaricomycetes (89.57%) and Tremellomycetes (10.14%) (Table S6). The combined abundance of the next 8 most abundant classes was less than 1% (Figure 2C, Table S6). At the species level, *S. hirsutum* and *N. aurantialba* were the dominant taxa (Figure 2B, Table S7), with both exhibiting significant differences in relative abundance between the basidioma and the substrate (Figure 3B). *S. hirsutum* exhibited the highest mean relative abundance (90.43%) across all 17 sampling points, with significantly higher abundance in the substrate compared with the basidioma. Within the basidiomata, *N. aurantialba*’s relative abundance was greater in central and basal regions than at the edges. In the substrate, the relative abundance of *S. hirsutum* showed a progressive increase with distance from the basidioma, reaching 95.52%, 98.82% and 99.18% in samples N06, N73 and N09, respectively (Figure 2D). Conversely, the relative abundance of *N. aurantialba* increased towards the periphery of the basidiomata, ranging from 14.39% to 27.46%. Its abundance in the substrate was minimal, accounting for less than 1% of the total fungal community, except near the inoculation point (N10). Whereas, its abundance decreased markedly to only 0.14%, 0.02% and 0.11% at the sampling points N71, N75 and N09, which were located furthest from the basidiomata interface (Figure 2D). These spatial distribution patterns suggest that *S. hirsutum* functions primarily as a structural component for the formation of JinEr basidiomata, rather than solely as a nutrient-providing host. This observation is in line with our previous research [30].

### 3.2. Community Diversity of the Endomycotic Microbiome

#### 3.2.1. Bacterial Diversity Within Basidiomata and Substrate Compartments

**Figure 4:**
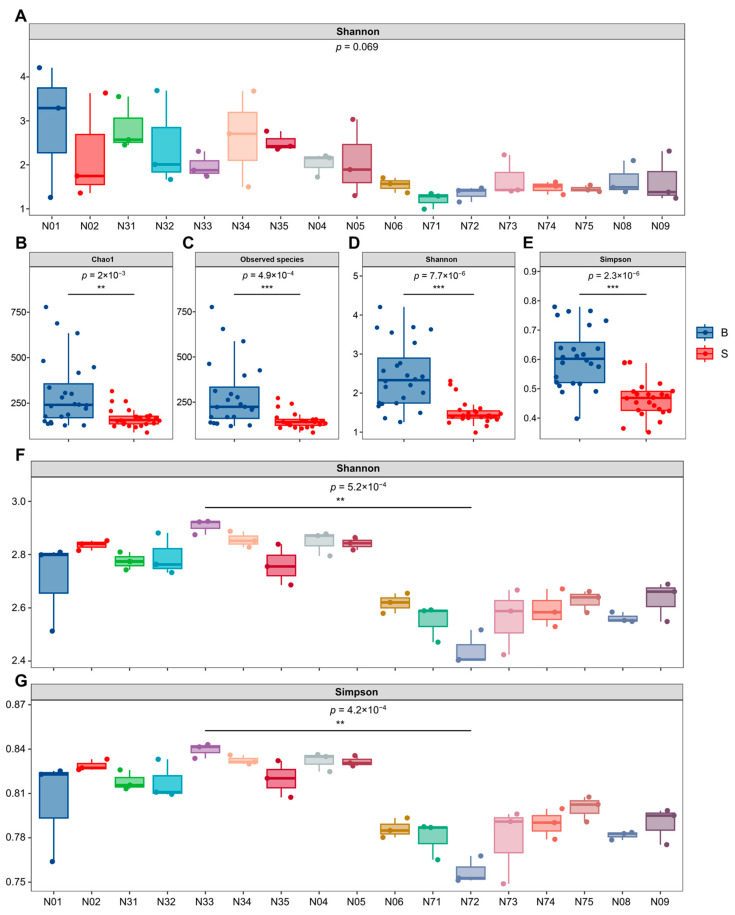
Endomycotic bacterial (**A**–**E**) and fungal (**F**,**G**) diversity comparisons. Microbial richness and diversity were assessed using the Chao1 index, Observed Species index, Shannon index, and Simpson index. Horizontal black bars spanning pairwise comparison denote differences identified by Student’s *t*-test. Asterisks indicate the statistical significance of mean differences (*** *p* < 0.001; ** *p* < 0.01). Basidiomata included samples N01, N02, N31, N32, N33, N34, N35, and N04. Similarly, the substrate included 8 compartments (samples N06, N71, N72, N73, N74, N75, N08, and N09) as illustrated in Figure 1.

To assess the influence of spatial compartments (17 sampling points) and fractions (basidiomata versus substrate) on endomycotic bacterial diversity, comparative analyses were conducted using four distinct diversity indices. Within basidiomata, endomycotic bacterial diversity, while numerically higher in peripheral compartments (e.g., N01, N31, and N35) relative to the central compartment (sample N33), exhibited no statistically significant difference (Figure S2). Conversely, within the substrate fraction, Shannon diversity indices for endomycotic bacteria were highly consistent across all compartments, indicating negligible spatial variation in diversity (Figure 4A). Consequently, spatial compartmentalization within either the basidiomata or substrate fractions had no significant effect on endomycotic bacterial diversity.

From a holistic perspective, endomycotic bacterial richness and diversity within basidiomata were both significantly greater than those within the substrate fraction. This elevated richness was evidenced by the Chao1 index and Observed Species index (Figure 4B,C), while increased diversity was confirmed by the Shannon and Simpson indices (Figure 4D,E). Therefore, tissue fraction (basidiomata versus substrate) was the principal determinant of endomycotic bacterial diversity.

#### 3.2.2. Community Diversity of Endomycotic Fungi

*S. hirsutum* and *N. aurantialba* collectively dominated the endomycotic fungal community, representing 99.67% of the total relative abundance. The remaining endomycotic fungi constituted an average relative abundance of 0.33%. Characterization of α-diversity using Shannon and Simpson indices revealed significantly higher endomycotic fungal diversity within the basidiomata compared to the substrate fraction. Maximum endomycotic fungal diversity was recorded in basidiomata sample N33, whereas the lowest diversity occurred in substrate sample N72. This difference in diversity was statistically significant (*p* < 0.01, Figure 4F,G). β-diversity analysis was performed using Non-metric Multidimensional Scaling (NMDS) coupled with PERMANOVA (sample size = 48, F = 114.34, *p* < 0.01, Figure S3). These results indicated the strong variation between fruiting body and substrate.

Collectively, differences in microbial diversity were notably observed between basidiomata and substrate fractions. Specific taxa, such as the prokaryotic genus *Delftia*, exhibited significant variation in abundance across different compartments.

### 3.3. Core and Keystone Bacteria in the Samples

**Figure 5:**
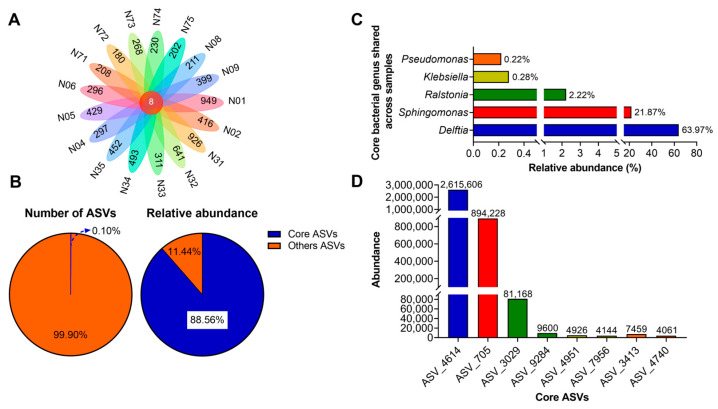
Core bacteriome analysis across all samples. (**A**) Venn diagram of core and unique bacterial ASVs across 17 sampling compartments. (**B**) Relative abundance and distribution patterns of the core bacteriome ASVs. (**C**) Taxonomic composition of the core bacteriome with their relative abundance in the whole dataset. (**D**) ASV-level composition of the core bacteriome, showing the top abundant core ASVs.

Further analysis identified core and unique microbial taxa shared across distinct compartments and fractions. Eight core bacterial ASVs were identified from a total pool of 8134 ASVs (Figure 5A). Notably, the number of unique endomycotic bacterial ASVs varied significantly between compartments. Among the 17 compartments analyzed, the number of unique bacterial ASVs ranged from 180 in sample N72 to 949 in sample N01. Within the basidiomata, the number of unique bacterial ASVs ranged from 297 in sample N04 to 949 in sample N01. Conversely, within the substrate fraction, unique bacterial ASVs ranged from 180 in sample N72 to 399 in sample N09.

Although these eight core bacterial ASVs constituted merely 0.10% of the total ASVs, they accounted for 88.56% of the total bacterial sequences across all 17 compartments (Figure 5B). These were therefore identified as potential core microbiome members (i.e., keystone bacteria). Taxonomic classification revealed these eight core ASVs belonged to five genera: *Delftia* (63.97%), *Sphingomonas* (21.87%), *Ralstonia* (2.22%), *Klebsiella* (0.28%), and *Pseudomonas* (0.22%) (Figure 5C). The most abundant ASV was ASV_4614 (unclassified *Delftia*), accounting for 2,615,606 sequences. This was followed by ASV_705 (unclassified *Sphingomonas*) accounting for 894,228 sequences, and ASV_3029 (unclassified *Ralstonia*) accounting for 81,168 sequences (Figure 5D, Table S8).

### 3.4. Functional Analysis of the Endomycotic Microbiome

**Figure 6:**
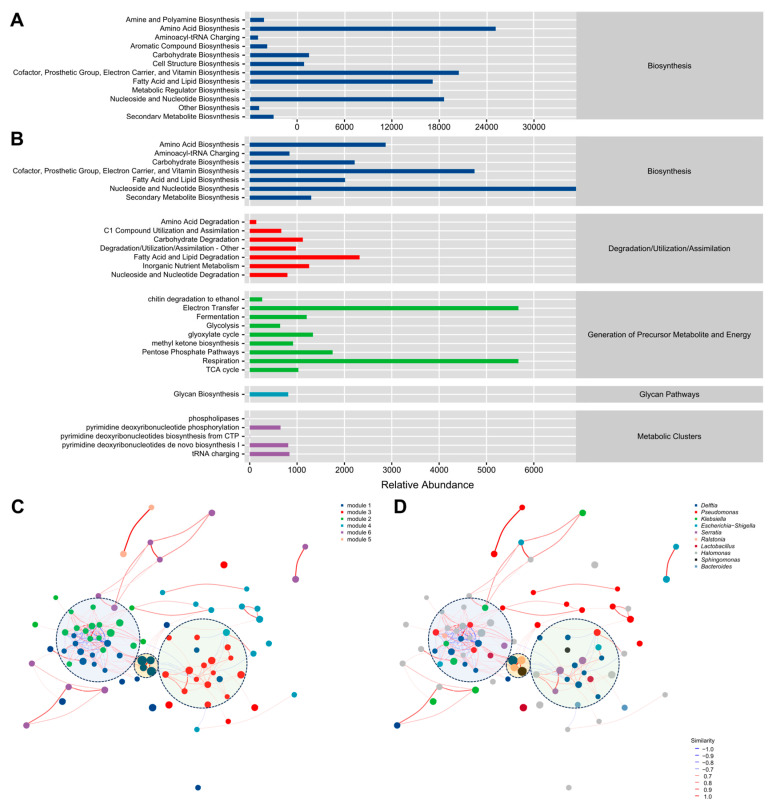
Functional profiling and network analysis of endomycotic communities. (**A**,**B**) Predicted functional abundance of endomycotic bacterial (**A**) and fungal (**B**) communities inferred from the MetaCyc genome database. The horizontal axis represents the relative abundance of the functional pathway at the second classification level, expressed as pathways (PWY) per million. Corresponding first-level pathway classifications are indicated adjacent to each bar. (**C**,**D**) Co-occurrence networks of endomycotic bacteria constructed using correlation matrices derived from Amplicon Sequence Variant (ASV) data. (**C**) Network organized by functional modules. (**D**) Network depicting the 100 most abundant bacterial genera. Nodes represent individual bacterial taxa. Edge colors denote the Pearson correlation coefficient (r) between nodes, spanning negative (blue; r < 0) to positive (red; r > 0) associations. Modules, defined as clusters of highly interconnected nodes, are distinguished by distinct colors.

To characterize the functional potential of the endomycotic microbiome, samples were clustered and the predominant functional categories of bacterial and fungal microbiota within the JinEr mushroom cultivation system were analyzed using the MetaCyc database. Functional prediction, inferred from genomic database mining, indicated significantly greater metabolic functional capacity within the bacterial microbiota compared with that of the fungal microbiota. Sixty-one functional pathways were grouped into seven principal functional categories: biosynthesis, degradation, detoxification, generation of precursor metabolite and energy, glycan pathways, macromolecule modification, and metabolic clusters (Figure 6A,B and Figure S4). Bacterial microbiota exhibited the highest inferred metabolic potential for biosynthesis of amino acids, cofactors, prosthetic groups, carriers, vitamins, fatty acid and lipid degradation, nucleosides, and nucleotides biosynthesis (Figure 6A). This suggests bacteria constitute a significant reservoir for secondary metabolism in the JinEr cultivation system. Within the fungal microbiota, nucleoside and nucleotide biosynthesis, electron transfer, and respiration were highly enriched (Figure 6B), which are primarily associated with physiological processes during the growth and development of *S. hirsutum* and *N. aurantialba*.

To further elucidate the ecological functional profile of the endomycotic bacterial microbiota, samples were clustered and the dominant ecological functional types in the JinEr cultivation system were analyzed using the FAPROTAX database. Moheterotrophy and aerobic chemoheterotrophy were the most predominant functional traits, which collectively account for over 85% of the total functional abundance (Table S9).

Co-occurrence network analysis was performed to identify key taxa and their relationships. This analysis revealed that bacterial taxa did not exist in isolation but formed interconnected co-occurrence clusters (Figure 6C,D). Modules 1 (blue), 3 (red), and 2 (green) exhibited substantial interconnectivity, forming the principal network modules (delineated by light color circles). No single bacterial genus exhibited pronounced dominance across the network. Associations between the genera varied in strength. Although *Delftia* and *Sphingomonas* did not display significant overall dominance within the network structure, they engage in extensive, predominantly positive interactions with numerous other bacterial genera.

### 3.5. Isolation and Identification of Culturable Endomycotic Bacteria

**Figure 7:**
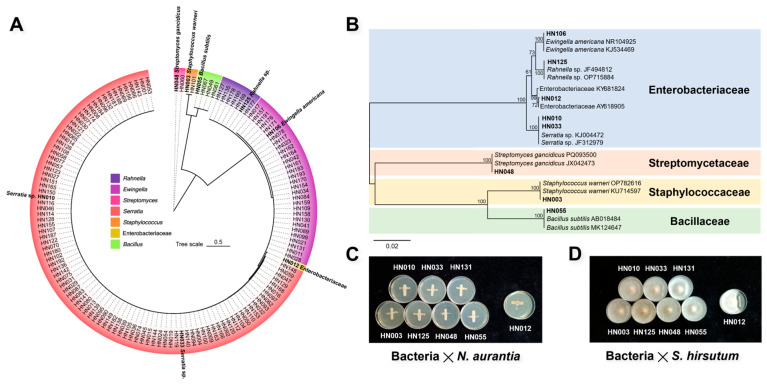
Culturable endomycotic bacteria from basidiomata. (**A**) Phylogenetic tree reconstructed from 16S rDNA sequences of 140 culturable endomycotic bacteria isolates. Isolates clustered into seven distinct phylogenetic groups: *Rahnella*, *Ewingella*, *Streptomyces*, *Serratia*, *Staphylococcus*, *Bacillus*, and a strain within the *Enterobacteriaceae* unidentified at the genus level. (**B**) Neighbor-Joining phylogenetic tree constructed using representative strains randomly selected from each of the seven groups. (**C**) Assessment of interactions between representative bacterial strains co-cultured with *N. aurantialba.* Fungal growth (*N. aurantialba*) is represented on the vertical axis and candidate representative bacterial strains are indicated on the horizontal axis. (**D**) Assessment of interactions between representative bacterial strains and *S. hirsutum*. *S. hirsutum* was centrally inoculated on the sterile Petri dish, while candidate representative bacteria were inoculated laterally along a designated line.

A total of 140 culturable endomycotic bacteria were obtained from the internal tissue of the basidiomata (Figure 7A). Phylogenetic classification based on 16S rDNA nucleotide sequences assigned these isolates to three bacterial phyla: Proteobacteria (representing 94.29% of total isolates), Firmicutes, and Actinobacteria. These isolates spanned four families: *Enterobacteriaceae*, *Streptomycetaceae*, *Staphylococcaceae*, and *Bacillaceae*. Seven genera were identified: *Rahnella*, *Ewingella*, *Streptomyces*, *Serratia*, *Staphylococcus*, and *Bacillus* (Figure 7A). The Proteobacteria constituted the dominant phylum, accounting for 94.29% of all isolates, with all members assigned to *Enterobacteriaceae*. Within the *Enterobacteriaceae*, *Serratia* was the most prevalent genus (90 isolates, 64.29%), followed by *Ewingella* (30 isolates, 21.43%) and *Rahnella* (7 isolates, 5.00%). Isolate HN012 could not be taxonomically resolved to the generic level (Figure 7B). Genera *Bacillus*, *Staphylococcus*, and *Streptomyces* collectively exhibited a combined frequency of approximately 5%.

One representative strain was randomly selected from each genus (with two representative strains from *Serratia*), yielding eight representative strains (HN003, HN010, HN012, HN033, HN048, HN055, HN106, and HN125; Figure 7B). These strains were subjected to phylogenetic reconstruction via neighbor-joining analysis (Figure 7B) and co-cultivated with *N. aurantialba* and *S. hirsutum*. Co-cultivation assays demonstrated that seven representative strains (excluding HN012) were capable of coexisting with both *N. aurantialba* (Figure 7C) and *S. hirsutum* (Figure 7D). The absence of antagonism observed between most endomycotic bacteria and their hosts thus indicates that these bacteria are not pathogenic microorganisms and exhibit compatibility with fungal host tissues.

## 4. Discussion

### 4.1. Potential Origins of Endomycotic Bacteria in JinEr Mushrooms

This study identified a diverse bacterial community within the basidiomata of JinEr mushrooms. Notably, bacteria of the genus *Delftia* spp. constituted the dominant taxon, exhibiting a relative abundance of 63.51% across the entire JinEr mushroom system. *Sphingomonas* spp. represented the second most abundant bacterial genus. Collectively, these two genera accounted for over 85% of the total microbial abundance. A statistically significant difference was observed in the relative abundance of genus *Delftia* between the basidiomata and the cultivation substrate.

Such dominance might involve the active recruitment of specific bacteria from the environment by the fungal host. Analogous to plants, which recruit beneficial microorganisms to enhance growth and confer protection against pathogens [31,32], fungi may employ similar strategies for hyphae and basidiomata development [11]. In cultivated edible mushrooms, bacteria are recognized for their critical roles in substrate conditioning, nutrient provisioning, growth stimulation, fruiting induction, and pathogen defense [11]. The pronounced dominance of *Delftia* and *Sphingomonas* suggests a highly specialized association with the JinEr mushroom basidiomata. This symbiosis may be the result of the JinEr mushroom recruiting its partner, or this association likely results from a reciprocal adaptation where the unique physicochemical environment of the basidioma (rich in specific polysaccharides and metabolites) creates a selective niche. Bacteria of these genera, equipped with versatile metabolic pathways, are particularly adept at colonizing and thriving in this niche. The specific mechanisms underlying this association, however, require further investigation and experimental validation. Microorganisms present in air or substrate can adhere to fungal surfaces and subsequently invade internal tissues, a phenomenon documented in both wild and cultivated mushrooms [12]. In this study, JinEr mushrooms were cultivated under sealed conditions using sterilized substrate inoculated with pure spawn of *N. aurantialba* and *S. hirsutum*. The high relative abundance of *Delftia* and *Sphingomonas* within the systems suggests these genera may be preferentially recruited. Airborne or waterborne bacteria within the cultivation environment could deposit on the basidiomata surface and be translocated systematically via the hyphal network. This selective recruitment and systematic distribution could potentially explain the observed lower bacterial density index within the cultivation bag compared to the basidiomata itself, indicating that the basidiomata might be the primary source of dominant bacteria within the entire system. Alternatively, the detected bacteria, or a subset thereof, may constitute endofungal bacteria (EFB) intrinsically associated with the host fungi (*N. aurantialba* and *S. hirsutum*). EFB, first identified in *Endogone* spores [33], typically colonize the host cytoplasm within mycelia [34,35] or spores [36]. EFB are prevalent across diverse fungal phyla, including Mucoromycota, Glomeromycota, Ascomycota and Basidiomycota [37,38]. Examples include *Paraburkholderia rhizoxinica* and *P. endofungorum* symbiotic with *Rhizopus microspores* [39]; the presence of Mollicutes-related bacteria within *Gigaspora margarita* hyphae was confirmed via electron microscopy and fluorescence in situ hybridization (FISH) [38], and *Chitinophaga* (Bacteroidetes) within the mycelium of *Fusarium keratoplasticum*, altering its carbon metabolism [40]. Furthermore, *Rhizobium* appears specifically associated with *Cantharellus cibarius* [18] and *Laccaria* spp. [19]. Given the significantly elevated relative abundance of *Delftia* and *Sphingomonas* across all samples in this study, it is hypothesized that these genera may represent EFB specific to JinEr mushroom. In conclusion, the dominant bacteria within JinEr basidiomata may originate either from active environmental recruitment or represent intrinsic EFB; distinguishing between these origins requires further investigation.

The genus *Delftia* was first established based on 16S rDNA sequence analysis [41]. It demonstrates significant biocontrol potential, exemplified by the endomycotic rice strain *D*. *tsuruhatensis* HR4 which inhibits pathogens including *Rhizoctonia solani*, *Pyricularia grisea*, *Xanthomonas oryzae* pv. *oryzae*, *Fusarium oxysporium*, and *Phytophthora melonis* [42]. This genus also exhibits notable capabilities in degrading organic pollutants and mediating cadmium transformations [43]. Prior to this study, reports of *Delftia* within macrofungi were scarce, suggesting its prevalence in JinEr mushrooms likely results from selective enrichment rather than stochastic colonization. Drawing parallels to plant systems, where beneficial keystone microbial taxa suppress disease through immune system priming, antibiotic production, and resource competition [44]. We hypothesize that *Delftia* may occupy an ecological niche by colonizing the basidiocarps of Auricularia auricula, thereby hindering the invasion of exogenous pathogens. Furthermore, it may potentially confer bioprotective effects by secreting certain antimicrobial substances [45,46].

The genus *Sphingomonas* exhibits ubiquitous distribution across diverse terrestrial and aquatic environments, including extreme environments [47]. These bacteria demonstrate significant tolerance to oligotrophic conditions and possess metabolic capabilities enabling the utilization of single molecules and the degradation of complex organic compounds. A primary catabolic focus of *Sphingomonas* involves polycyclic aromatic hydrocarbons and heterocyclic derivatives. Given that lignin represents one of the most abundant aromatic polymers in the biosphere [48], the presence of *Sphingomonas* within JinEr mushroom substrate may facilitate lignin decomposition. This activity could potentially enhance substrate utilization efficiency by *S. hirsutum*, thereby promoting basidiomata development. Furthermore, *Sphingomonas* strains synthesize secondary metabolites, including β-carotene and carotenoids [49,50,51], which function as antioxidants and serve as vitamin A precursors. These compounds possess documented immunomodulatory properties, and may impede cancer progression. Consequently, *Sphingomonas* colonization could contribute to the enhanced nutritional and commercial value of JinEr mushroom. Additionally, numerous *Sphingomonas* species exhibit ecosystemic functional attributes such as biological nitrogen fixation, phosphate solubilization, and production of phytohormones [52,53], potentially stimulating JinEr mushroom growth. Considering the prevalence of *Sphingomonas* in air and water, their detection is likely attributable to atmospheric deposition onto JinEr basidiomata surfaces, followed by selection, colonization, and dissemination throughout the cultivation environment. This colonization may constitute a significant factor in JinEr mushroom development [54,55].

A striking finding of this study was the extreme dominance of two bacterial genera, *Delftia* and *Sphingomonas*, which together constituted over 85% of the prokaryotic sequences within the JinEr mushroom basidiomata. This pronounced dominance within a community that otherwise harbored substantial taxonomic richness (1109 bacterial ASVs) presents a classic “low-evenness, high-richness” structure, a pattern not uncommon in host-associated microbiomes [56]. This pattern is ecologically plausible and underscores the highly selective nature of the mushroom fruiting body as a specialized microenvironment [24,57]. Such skewed community composition, where a few key taxa achieve decisive fitness advantage, is frequently observed in other closed, nutrient-rich symbiotic systems, such as insect guts [58] and root nodules [59]. The co-dominance of these two genera in our system suggests they may occupy complementary functional niches or engage in a synergistic relationship with the host fungus, potentially related to nutrient mobilization, growth promotion, or defense [9,60]. This may support the principle that in specialized symbiotic habitats, community function and structure are often driven by a few highly adapted symbionts rather than by sheer taxonomic diversity [56,61].

### 4.2. Potential Functions of Endomycotic Bacteria in JinEr Mushrooms

Bacterial α-diversity revealed significant diversity within the substrate compared to that in JinEr basidiomata. This disparity suggests that the endomycotic bacterial community within the substrate predominantly originates from the basidiomata. Although statistical significance was not attained, the Shannon diversity index was consistently higher in the peripheral regions (samples N01, N31, and N35) of the basidiomata relative to the internal regions (samples N02, N33, and N04). This pattern implies either microbial ingress from the external environment during basidiomata morphogenesis or active recruitment of beneficial bacteria (e.g., *Sphingomonas*) by *N. aurantialba* for internal colonization.

Core microbiome analysis identified eight bacterial ASVs co-occurring across all 17 sampled sites, exhibiting relative abundance trends congruent with the overall community profile. The genera *Delftia* and *Sphingomonas* consistently demonstrated the highest relative abundances. Co-occurrence network analysis indicated that endomycotic bacteria do not exist in isolation but form interconnected clusters. Despite their dominance within the community, *Delftia* and *Sphingomonas* did not exhibited pronounced centrality within the network topology. Instead, they engaged in predominantly positive interactions with a broad spectrum of other bacterial genera.

To further investigate the potential functional roles of endomycotic bacteria, we conducted functional profiling of the collective endomycotic bacterial community using the MetaCyc genome database. This analysis revealed biosynthesis pathways for amino acids, cofactors, prosthetic groups, carriers, vitamins, nucleosides, and nucleotides as the predominant metabolic category. Although predictive in nature, these findings indicate that the endomycotic bacterial microbiota constitutes a significant reservoir of secondary metabolic potential.

Within the JinEr mushroom cultivation system, chemoheterotrophy and aerobic heterotrophy represent the dominant ecological functions of the endomycotic bacterial community. Chemoheterotrophic bacteria derive energy and carbon through the catabolism of organic compounds. The cultivation environment, encompassing both substrate and basidiomata, provides an abundant source of organic matter, rendering chemoheterotrophy an efficient strategy for energy acquisition. Furthermore, the prevailing aerobic conditions within the substrate and basidiomata established an optimal environment for aerobic heterotrophic bacteria. Crucially, aerobic metabolism processes exhibit greater bioenergetic efficiency compared to anaerobic alternatives, providing a mechanistic basis for the dominance of these functional groups within the community.

### 4.3. Interaction Among S. hirsutum, N. aurantialba and Associated Microorganisms

Analysis of fungal ASV relative abundance revealed that the hyphae of *S. hirsutum* were predominantly localized within the substrate, where *N. aurantialba* representation was minimal. Even within the basidiomata, *S. hirsutum* accounted for more than 80% of fungal sequences, compared to <20% for *N. aurantialba*. Consequently, the cultivated JinEr mushroom is primarily composed of *S. hirsutum.* Given *N. aurantialba*’s limited capacity to decompose complex substrates such as crude fiber, and considering the pervasive distribution and primary role of *S. hirsutum* in substrate degradation, *S. hirsutum* functions as the principal nutrient provider within the symbiotic system [62]. In contrast, *N. aurantialba* adopts a parasitic relationship with *S. hirsutum*, deriving nutrients via absorption from its hyphae, ultimately co-forming the heterogeneous basidiomata structure [3,4].

The tolerance of *S. hirsutum* towards parasitism by *N. aurantialba* suggests a potential reciprocal exchange of benefits. *N. aurantialba* secretes diverse polysaccharides and secondary metabolites [3]. It is therefore hypothesized that *N. aurantialba* supplies *S. hirsutum* with growth-essential polysaccharides and secondary metabolites, facilitating its parasitic access to nutrients derived from *S. hirsutum.* Furthermore, the nutrient-rich microenvironment associated with *N. aurantialba* within the symbiotic system may recruit beneficial bacteria from the environment, potentially contributing to the growth and development of *S. hirsutum*.

### 4.4. Culturable Endomycotic Bacteria in JinEr Mushrooms

A total of 140 culturable bacterial strains, representing seven distinct genera, were isolated from JinEr mushroom basidiomata. Numerous isolates belonged to the genera Rahnella, Serratia and Staphylococcus, which are widely characterized as plant growth-promoting rhizobacteria possessing plant growth-beneficial potential [63,64,65]. Serratia constituted the fifth most abundant genus within the endomycotic bacterial community. Certain Serratia species demonstrated hyphal migration capacity and exhibited antagonistic effects against pathogenic fungi, suggesting potential utility in agricultural fungal biocontrol [66,67]. Genomic annotation and comparative analyses have identified conserved and strain-specific genes associated with nitrogen fixation, phytohormone synthesis, organic acid biosynthesis, and the production of volatile organic compounds (VOCs) in several Rahnella strains, conferring direct plant growth-promoting benefits [63,68]. Additionally, specific isolates demonstrated bioprophylactic activity, potentially protecting the host from fungal pathogens [69]. Certain Streptomyces species are known to enhance basidiomata production and contribute to disease control against pathogens causing green mold disease and mushroom blotch [70,71]. Bacillus species, prevalent in mushroom habitats, exhibit potential as biocontrol agents [72,73]. Conversely, members of the genus Ewingella (notably *E. americana*) are more frequently documented as mushroom pathogens [74,75]. Collectively, the isolated culturable bacteria exhibit diverse functional potentials, with the majority demonstrating symbiotic compatibility with the host fungus without significant antagonism. These isolates represent candidates for subsequent development as mushroom growth-promoting agents. Notably, the isolated culturable bacteria did not correspond to dominant populations based on ASV relative abundance. This discrepancy indicates that the majority of endomycotic bacteria within JinEr mushroom basidiomata are not readily culturable under the employed conditions.

A clear discrepancy was observed between the molecular and cultivation-based analyses: *Delftia* and *Sphingomonas* dominated the amplicon sequencing profiles, whereas cultivation on standard nutrient-rich media primarily recovered fast-growing genera such as *Staphylococcus*. This divergence likely originates from a media selection bias, suggesting that the predominant endomycotic taxa may thrive under oligotrophic conditions not provided by the rich media used. Other contributing factors could include challenging physiological states (e.g., viable but non-culturable, VBNC) among key community members. The isolation of these fast-growing genera, some of which include opportunistic pathogens, underscores the importance of stringent hygiene in cultivation systems. Future studies should employ oligotrophic media and extended incubation to target the currently uncultivated majority, thereby better aligning isolation outcomes with molecular community data.

This study focused exclusively on isolating and characterizing culturable endomycotic bacteria, guided by an applied goal of identifying plant-growth-promoting candidates for mushroom cultivation. The fungal endomycotic community was not isolated, as fungal cultures generally present greater challenges in scale-up and may introduce competitive interactions with the host. Future work expanding to include fungal partners would offer a more comprehensive understanding of the total symbiotic microbiome.

## 5. Conclusions

This study represents the first systematic investigation of the microbial diversity associated with the JinEr mushroom and its cultivation substrate, yielding novel insights into their microbial ecology. We found that the symbiotic system associated with the JinEr mushroom cultivation exhibits greater complexity than recognized. Quantitative analysis established *S. hirsutum* as the predominant constituent of the biomass of this edible and medicinal mushroom. Community profiling identified *Delftia* and *Sphingomonas* as the dominant endomycotic bacterial genera in the basidiomata. Representatives of both genera are likely to be established as stable endofungal bacteria within the fungal host. Marked disparities in endomycotic bacterial diversity were observed between the basidiomata and the cultivation substrate. Notably, alpha diversity indices were significantly elevated in the basidiomata relative to the substrate, suggesting potential translocation of environmental or endofungal bacteria from the basidiomata into the substrate. Culturing efforts yielded 140 cultivable bacterial isolates (7 genera, 4 families) from the basidiomata. Subsequent in vitro coculture assays demonstrated pronounced compatibility between the majority of isolated strains and the host fungus. Collectively, these findings advance our understanding of JinEr microbiome dynamics, contribute to elucidating the symbiotic relationship (whether mutualistic or parasitic), and establish a foundation for the targeted isolation and application of beneficial bacterial consortia to optimize cultivation systems.

## Data Availability

The data presented in this study are openly available in NCBI at https://www.ncbi.nlm.nih.gov/ (accessed on 31 December 2024), reference number PRJNA1204465.

## Supplementary Materials

The following supporting information can be downloaded at: https://www.mdpi.com/article/10.3390/jof12010041/s1, Figure S1: Rarefaction curves of detected bacterial (A) and fungal (B) Chao1 index of the 17 compartments reach saturation stage with increasing flattening depth.; Figure S2: Differences in bacterial diversity between the edges and interiors of fruit body. Figure S3: Samples’ endophytic fungal community compositions as indicated by non-metric multidimensional scaling plots (NMDS) of pairwise Bray–Curtis distance. Abbreviations: F: fruiting body, includes 8 compartments (sample N01, N02, N31, N32, N33, N34, N35, N04); S: substrate, includes 8 compartments (sample N06, N71, N72, N73, N74, N75, N08, N09). Figure S4: Endophytic bacterial community functional abundance prediction based on MetaCyc genome database. The horizontal axis is the relative abundance of the functional pathway (unit is the PWY (pathways involved in metabolism) per million) of the second classification level of MetaCyc, and to the right is described the first-level pathway to which this functional pathway belongs. Table S1: Taxonomic composition and raw read counts of endomycotic bacteria; Table S2: Taxonomic composition and raw read counts of endomycotic fungi; Table S3: Relative abundance of top 10 endomycotic bacterial phyla found in the samples; Table S4: Relative abundance of top 10 endomycotic bacterial genus found in the samples; Table S5: Relative abundance of top 10 bacterial genus found in the samples; Table S6: Relative abundance of top 10 fungal class found in the samples; Table S7: Relative abundance of top 10 fungal species found in the samples; Table S8: Core bacterial ASVs of fruiting bodies; Table S9: Endomycotic bacterial community functional abundance prediction based on FAPROTAX database.
