# Supplementary material for: Symbiosis Among Naematelia aurantialba, Stereum hirsutum, and Their Associated Microbiome in the Composition of a Cultivated Mushroom Complex JinEr
Source: J Fungi (Basel). 2026 Jan 4;12(1):41. doi: 10.3390/jof12010041 (PMC12843488; doi:10.3390/jof12010041)
Supplement: Supplementary file 1 [file jof-12-00041-s001.zip › Supplementary Figures.pdf]

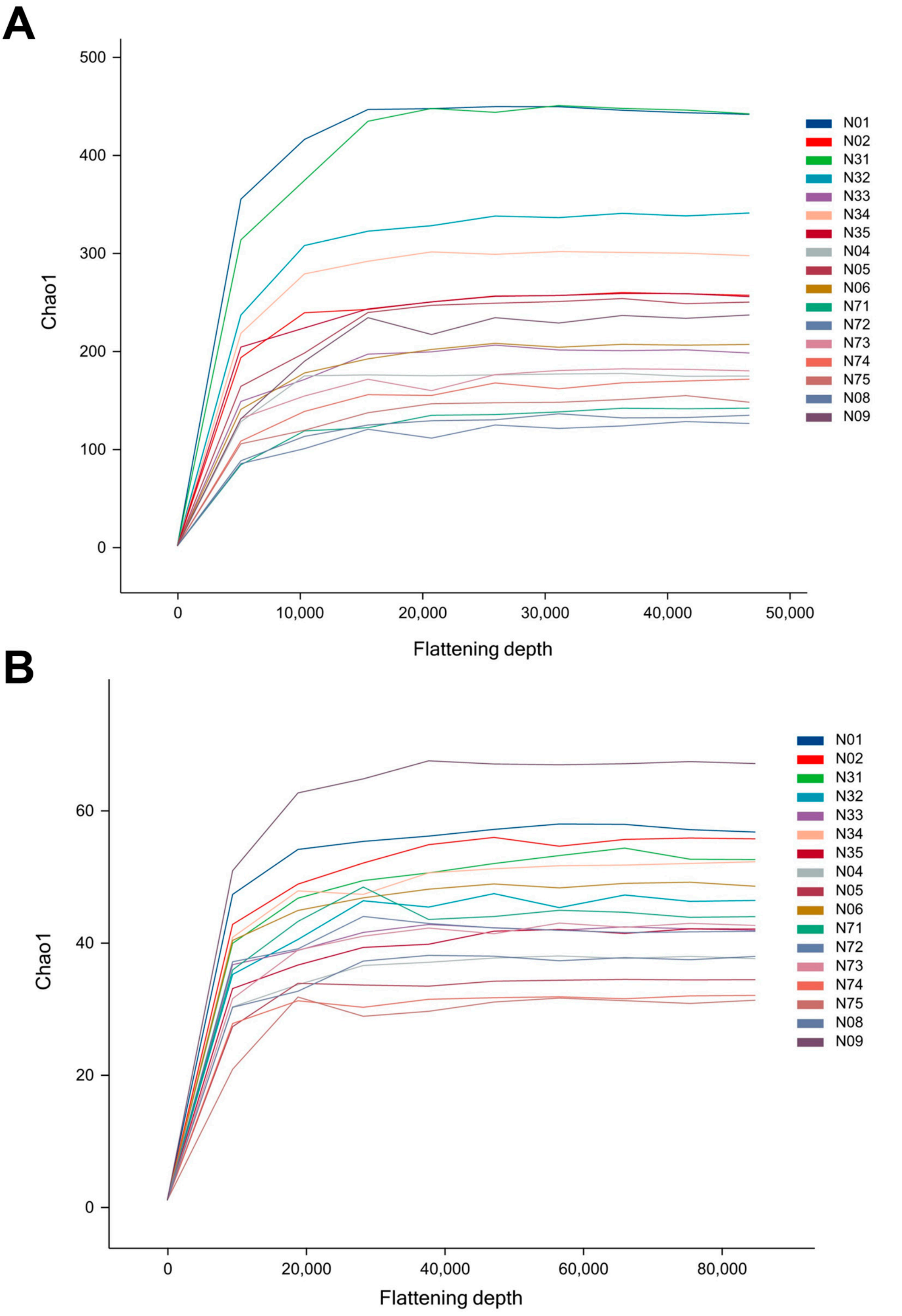

Figure S1. Rarefaction curves of detected bacterial (A) and fungal (B) Chao1 index of the 17 compartments reach saturation stage with increasing flattening depth.

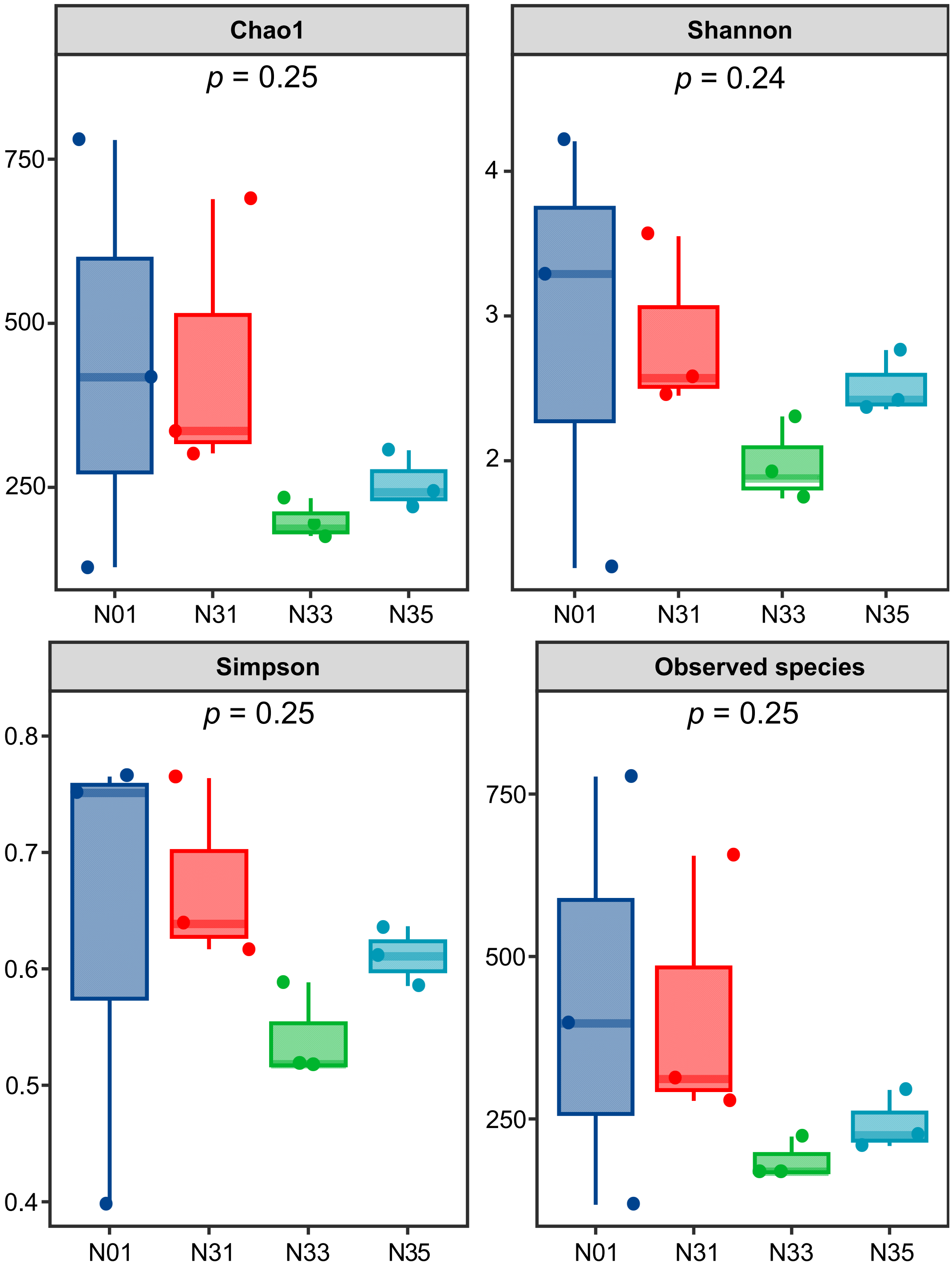

Figure S2. Differences in bacterial diversity between the edges and interiors of fruit body.

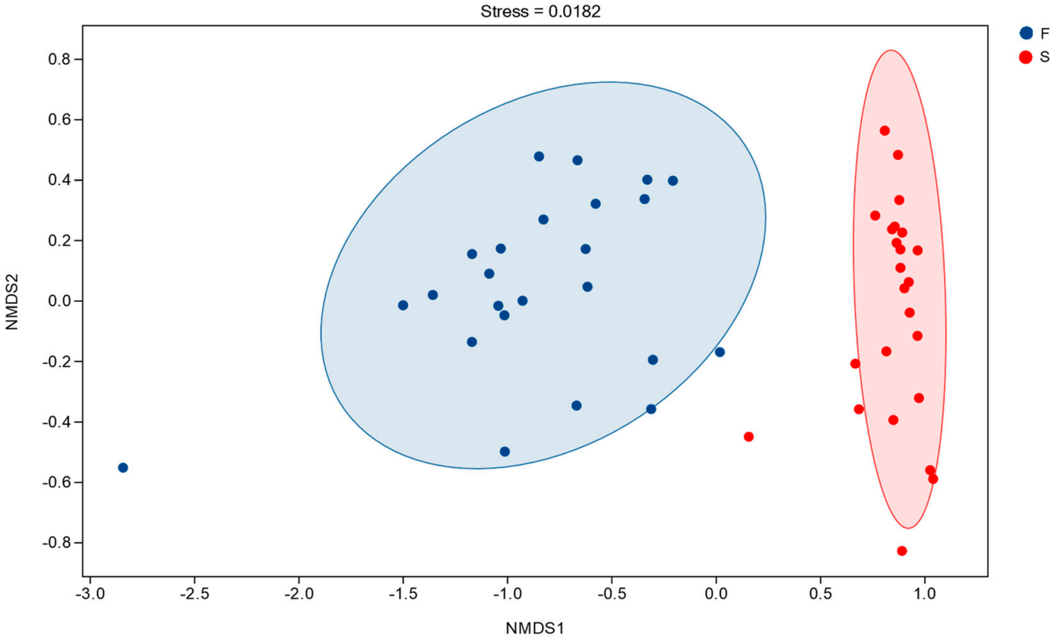

Figure S3. Samples' endophytic fungal community compositions as indicated by non-metric multidimensional scaling plots (NMDS) of pairwise Bray-Curtis distance. Abbreviations: F: fruiting body, includes 8 compartments (sample N01, N02, N31, N32, N33, N34, N35, N04); S: substrate, includes 8 compartments (sample N06, N71, N72, N73, N74, N75, N08, N09).

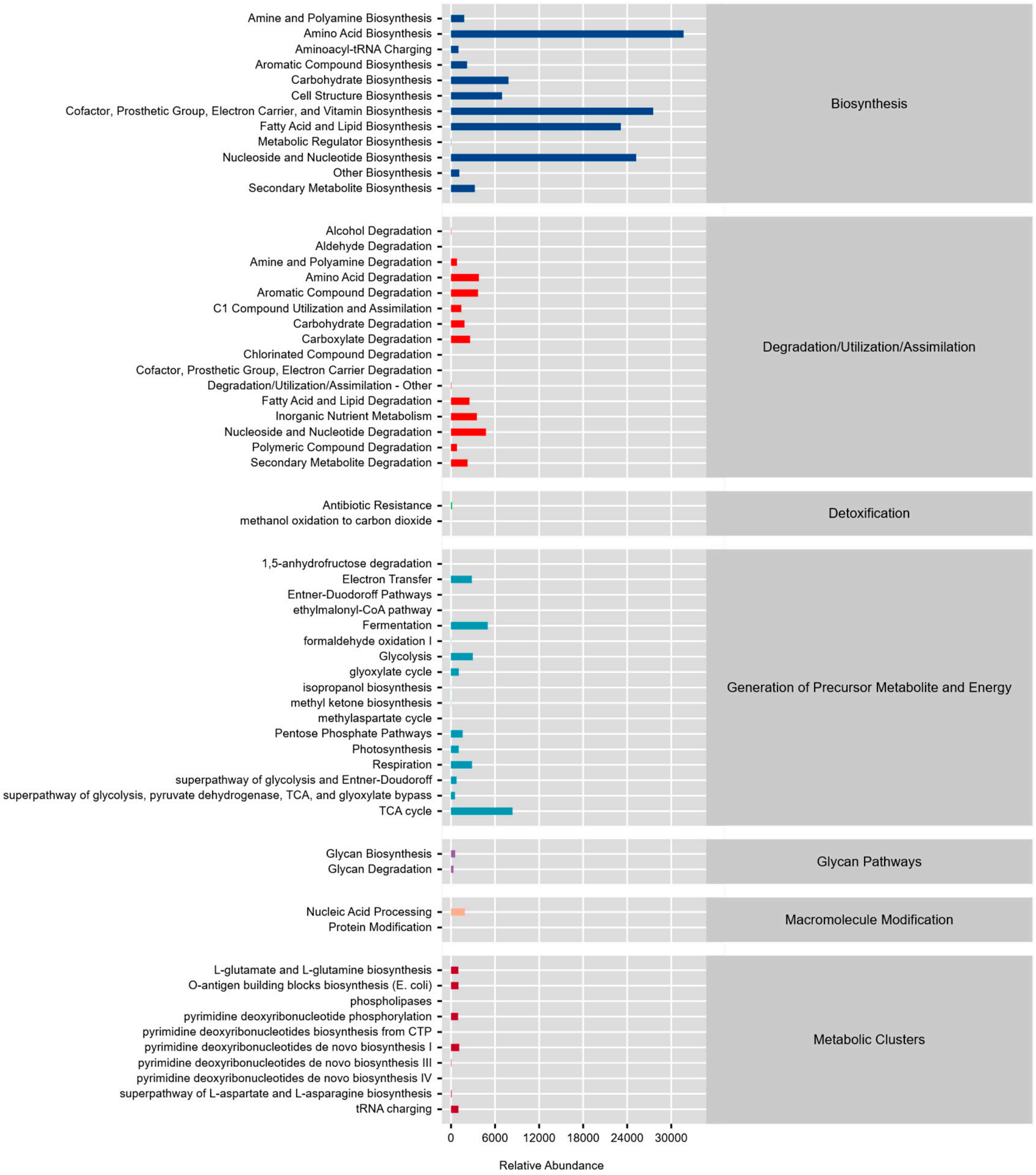

Figure S4. Endophytic bacterial community functional abundance prediction based on MetaCyc genome database. The horizontal axis is the relative abundance of the functional pathway (unit is the PWY (pathways involved in metabolism) per million) of the second classification level of MetaCyc, and to the right is described the first-level pathway to which this functional pathway belongs.
